# Reactive oxygen species associated immunoregulation post influenza virus infection

**DOI:** 10.3389/fimmu.2022.927593

**Published:** 2022-07-29

**Authors:** Lan Wang, Zheng Cao, Zi Wang, Jimin Guo, Jing Wen

**Affiliations:** ^1^ Department of Microbiology, Immunology and Molecular Genetics, David Geffen School of Medicine, University of California Los Angeles (UCLA), Los Angeles, CA, United States; ^2^ UCLA Acquired Immune Deficiency Syndrome (AIDS) Institute, University of California Los Angeles (UCLA), Los Angeles, CA, United States; ^3^ Department of Chemical and Biomolecular Engineering, University of California Los Angeles (UCLA), Los Angeles, CA, United States

**Keywords:** reactive oxygen species, influenza virus, viral replication, innate immune response, adaptive immune response

## Abstract

An appropriate level of reactive oxygen species (ROS) is necessary for cell proliferation, signaling transduction, and apoptosis due to their highly reactive character. ROS are generated through multiple metabolic pathways under a fine-tuned control between oxidant and antioxidant signaling. A growing number of evidence has proved their highly relevant role in modulating inflammation during influenza virus infection. As a network of biological process for protecting organism from invasion of pathogens, immune system can react and fight back through either innate immune system or adaptive immune system, or both. Herein, we provide a review about the mechanisms of ROS generation when encounter influenza virus infection, and how the imbalanced level of ROS influences the replication of virus. We also summarize the pathways used by both the innate and adaptive immune system to sense and attack the invaded virus and abnormal levels of ROS. We further review the limitation of current strategies and discuss the direction of future work.

## Introduction

Reactive oxygen species (ROS) are a class of partially reduced metabolites of oxygen (O_2_). There are two categories, radical and non-radical, of ROS based on the number of unpaired electrons in their outmost shell (1). ROS is highly reactive and can oxidize intracellular macromolecules in response to endogenous and/or exogenous stimuli, which makes them crucial elements for cellular activities (2). The production of ROS is fine-tuned by oxidant and antioxidant mechanisms under physiological metabolism. An appropriate level of ROS is necessary for cellular processes, such as cell growth, signaling transduction, and apoptosis (3). It is believed that excessive accumulation of ROS leads to aggravation of inflammation, augment of protease secretion, and accumulation of ROS intermediates, which ultimately resulting in inflammation response, apoptosis, and tissue injury (4). Evidence has proved that ROS have served as a crucial contributor to viral disease. This review focuses on the impact of ROS on viral replication and immune response during influenza virus (IV) infection.

## ROS production after IAV infection

**Figure 1:**
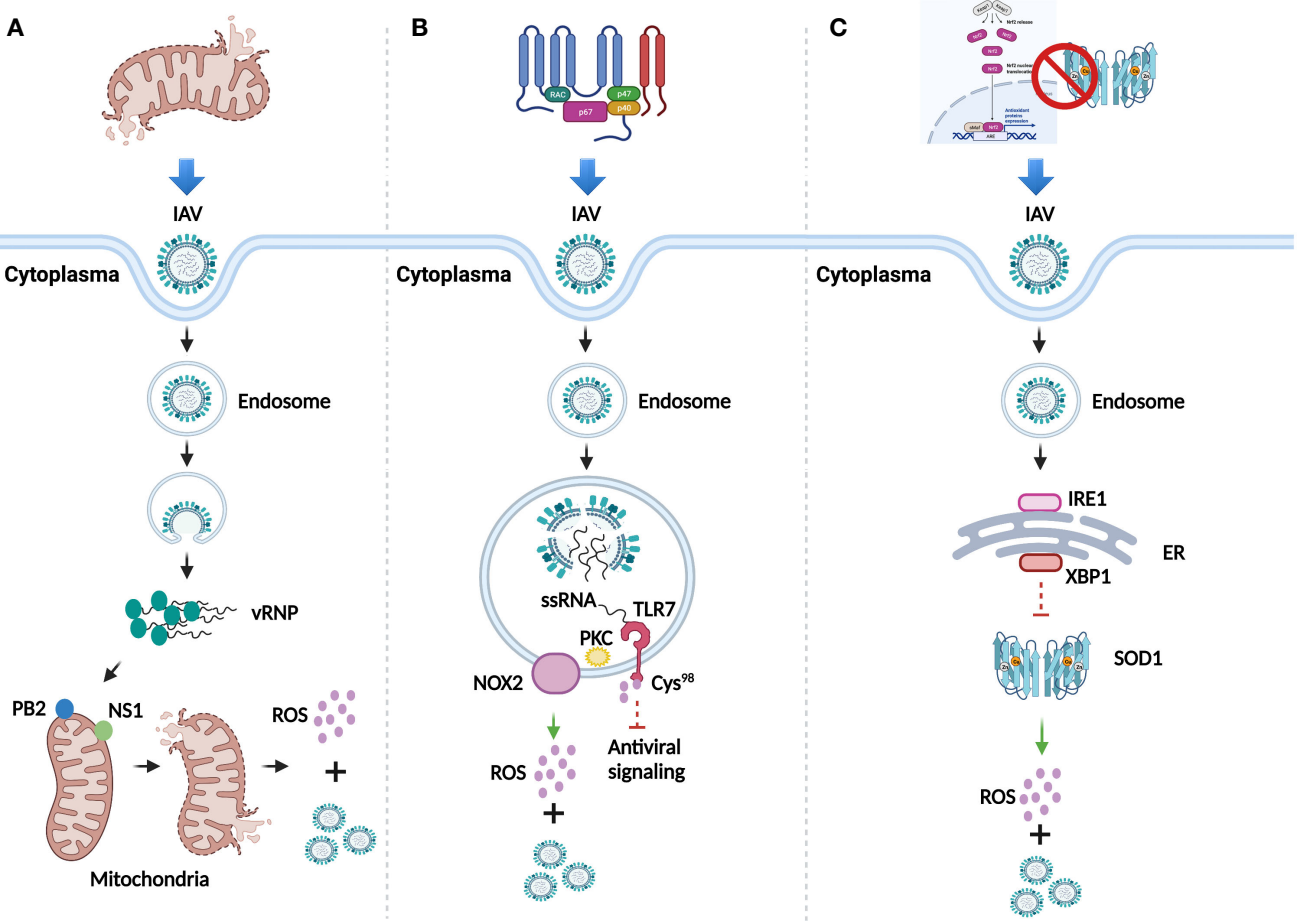
ROS generation after IAV infection. **(A)** Dysfunction of mitochondria mediated by IAV infection for the generation of ROS and viral replication. **(B)** ROS generation and viral replication mediated by activation of NADPH oxidase. **(C)** Inhibition of antioxidant signaling pathway and antioxidant enzymes for ROS generation and viral replication.

IV is a family of negative-sense single-stranded RNA virus which belongs to *Orthomyxoviridae*. It can be classified into four major genera, including *Alphainfluenzavirus* (*Influenza A virus*, IAV), *Betainfluenzavirus* (*Influenza B virus*, IBV), *Gammainfluenzavirus* (*Influenza C virus*, ICV), and *Deltainfluenzavirus* (*Influenza D virus*, IDV). Since IAV is responsible for the majority of seasonal epidemic or pandemic threat to human (5, 6), this review focuses on the effect of ROS induced by IAV infection. Overall, dysfunctional mitochondria, activated nicotinamide adenine dinucleotide phosphate oxidase (NADPH oxidase), and inhibited antioxidant signaling pathway and enzymes, are three main mechanisms involved in the multi-coordinated process of ROS production post IAV infection (7) (**Figure 1**).

### Dysfunction of mitochondria

Mitochondria, the powerhouse of the cells, can generate ROS through respiration. Viral polymerase is responsible for viral genome replication and transcription in the nuclei of host cells (8). IAV-dependent RNA polymerase complex contains a subunit, the polymerase basic 2 (PB2) protein, which can import IAV into the matrix space of mitochondria due to its N-terminal mitochondrial targeting sequence. Accumulation of IAV in the mitochondria breaks the equilibrium of mitochondrial fusion and fission, causing their fragmentation (9) and leading to the leak of superoxide ion into the cytoplasm (10). Besides PB2, the nonstructural protein 1 (NS1) can also induce fragmentation of mitochondria by altering its dynamics (11), which indicates the stimulation role of NS1 in ROS production. An increased level of ROS promotes the replication of IAV to enhance its pathogenesis (12) (**Figure 1A**).

### Activation of NADPH oxidase

NADPH oxidase also known as NOX, is a membrane-bound complex present in cells and phagosomes. Human NOX isoforms comprise NOX1 to NOX5, dual oxidase 1 (DUOX1), and DUOX2 (13). Evidence has proved that ROS significantly increase in lung epithelial cell lines and primary murine cells in a NOX4-dependent manner after IAV infection (14). Besides, observed in primary murine macrophages, endosomal NOX2-induced ROS generation was mediated by the toll-like receptor 7 (TLR7) and subsequent activation of protein kinase C (PKC), while the generated ROS further suppressed the antiviral signaling through the modification of cysteine residue Cys^98^ of TLR7 to support viral proliferation (15) (**Figure 1B**). Inhibition of NOX2 significantly attenuated neutrophil influx, alveolitis, and ROS generation from inflammatory cells in IAV infected C57Bl/6 mice, which confirmed the role of NOX2 oxidase for promoting ROS generation (16). Additionally, the presence of IAV promotes the translocation of p67-phox, a cytosolic protein of NOX, to the cell membrane for ROS production at the early infection stage (17).

### Inhibition of antioxidant signaling pathway and antioxidant enzymes

The Nrf2/Keap1 pathway is the principal antioxidant signaling cascade for protecting against ROS stress. Evidence has showed that in isolated human alveolar type II (ATII) cells and alveolar macrophages (AM), IAV infection increases the level of ROS and translocation of Nrf2 to the nuclei, whereas overexpression of Nrf2 decreases viral replication and oxidative stress (18). In addition, downregulated Nrf2 and upregulated ROS were observed in both IAV infected primary normal human bronchial epithelial (pNHBE) cells and BALB/c mice with IAV infection (19). Besides antioxidant signaling pathway, ROS can also be neutralized by antioxidant enzymes, such as superoxide dismutase (SOD), catalase (CAT), and glutathione peroxidase (GPx). The investigation of IAV infection in human alveolar cells has showed that the loss of copper-zinc SOD1 contributed to the increased level of superoxide anion and viral replication (20). A study has further disclosed the underlying mechanism that endoplasmic reticulum (ER)-associated degradation (ERAD) regulates the redox state to potentiate IAV infection (21). The presence of IAV first stimulated inositol-requiring 1(IRE1), a core sensor of ER stress signaling pathway, then IRE1 activated downstream factor X-box binding protein-1 (XBP1) through mRNA splicing, which could further promote ERAD. The whole process is likely to be responsible for the reduction of SOD1, thus leading to the increase of ROS and viral replication (**Figure 1C**).

### Other mechanisms

Apart from the three main mechanisms, other mechanisms of IAV to produce ROS and favor its replication have been proposed. In human lung cancer A549 cells, decreased sirtuin 2(SIRT2) reduces the expression and activity of glucose-6-phosphate dehydrogenase (G6PD) by acetylation, resulting in enhanced production of ROS and IAV replication (22). The binding between aryl hydrocarbon receptor (AhR) and its ligand, quinone 1 (NQO1) is also reported to involve in the production of ROS (5, 23, 24). Additionally, neutrophil is another major source of ROS either *via* a neutrophil elastase (NE) mediated mechanism (25) or myeloperoxidase (MPO) enzyme (26) to assist pathogen clearance.

## The immune response mediated by ROS responding to IAV infection

The innate and adaptive immune systems are critical for pathogen-specific defense. Substantial evidence has revealed that ROS are essential messengers in immune cells. The balanced production and elimination of ROS maintains a healthy immune system in physiological situation. The presence of virus induces increased levels and disturbs the balance of ROS within immune cells, resulting in activation of both innate and adaptive immune response. Additionally, ROS not only work as critical components of the host to fight against the invading virus, but also possess significant role to transmit signals from multiple signaling pathways to regulate the phenotype and function of immune cells (27). However, accumulated ROS in pathological condition may persistently stimulate the immune system and induce hyperactivation of inflammatory responses, resulting in tissue damage and pathology (28, 29).

### Innate immune response

#### Inflammasome

Inflammasome pathway enables the detection of pathogens, release of cytokines, and recruitment of effector cell to the infection site (30). It shows that stimulation of NF-κB activates NLRP3 inflammasome (31). IAV virulence protein, PB1-F2, can activate NLRP3 inflammasome as well, but also impaired its activation *via* various mechanisms (31). Besides, mitochondrial-derived ROS are proved to promote the activity of NLRP3 inflammasome (32, 33). This activation could further drive the expression of interleukin-1β (IL-1β) (33). A novel MxA inflammasome has also been reported to be involved in IAV mediated immune response (34). In respiratory epithelial cells, MxA recognizes the nucleoprotein of IAV, then interacts with ASC to release IL-1β through the activation of caspase 1 (35).

#### Neutrophils

Neutrophils are first responders to be recruited to the site of infection and recognize IAV through TLR7/8 (36). A mode used by neutrophils for their recruitment in lung microenvironment is due to the induction of chemokine receptors (37). By using single-cell RNA sequencing assay, a study shows that PD-L1^+^ neutrophils are the major contributor for releasing pro-inflammatory factors in the first 1-3 days post infection (38). The function of neutrophils during IAV infection is controversial (39). A protective role is found to inhibit viral spread based on mice data that, early recruitment of neutrophils and their derived chemokine CXCL12 were essential for the migration of CD8^+^ T-cells to infected trachea (40). Another report proved that CD11b/CD18 integrin (MAC-1) helped neutrophil to suppress IAV-induced, T-cell mediated pathology probably by restraining the proliferation of T-cells (41). Neutrophil-derived secretion of IL-1β is thought to protect against virus. By releasing peptide mCRAMP, NLRP3 inflammasomes in alveolar macrophages were activated by neutrophils, which led to the release of IL-1β to combat infection (42). However, hyperactivated neutrophils with excessive recruitment, robust inflammatory reaction, and neutrophil extracellular traps (NETs) (39), can induce sever lethal effect in the acute respiratory distress syndrome (ARDS) caused by IAV infection (43). NETs contain histone and granule proteins, their formation relies on neutrophil produced ROS, NE and MPO (44). Evidence has demonstrated that high levels of NETs are correlated with poor prognosis of severe IAV infection and fatality in patients (45, 46).

#### Macrophages

Alveolar macrophages (AMs) are the most relevant macrophages for initiating inflammatory and immune responses to IAV infection in the lung. An animal study shows that a group of platelet factor 4-positive (Pf4^+^)-macrophages, probably the precursors of AMs, can generate pro-inflammatory factors 7 days after IAV infection (38). AMs contribute to protect alveolar epithelial cells (AECs) from IAV infection., The cysteinyl leukotriene (CysLT) trigged pathway is suppressed by AMs, which is considered to prevent AECs damage from the virus (47). The protective role of AMs during IAV infection might be due to their peroxisome proliferator-activated receptor gamma (PPAR-γ). Activation of PPAR-γ ameliorates virus-associated inflammation and increases the level of MMP7 and MMP9, tissue remodeling factors, and EGF and VEGF, epi-endothelial growth factor, for repair of damaged sites (48, 49). The impact of ROS on macrophages against IAV infection, as well as ROS mediated signaling pathways in infected macrophages, are indirect (50). Evidence has showed that the polarization of M1 macrophages depends on ROS mediated pathway (51); in M2 macrophages, ROS either promote their polarization *via* NF-κB (52), or induce autophagy to inhibit their polarization *via* MAPK pathway (53). Besides, ROS destroy exogenous materials in the antigen presenting route to reduce the antigen presentation of macrophages (54). Furthermore, ROS mediated autophagy strengthens the formation of MHC class II for macrophages (55). These data indicates that ROS can impact the function of macrophages, but further investigation is needed to fully understand the effects of IAV infection on ROS-influenced macrophages.

#### Natural killer cells

NK cells are recruited in the lung within the first few days following IAV infection in mice (56). The presence of IAV activates NK cells through STAT4 to secrete IFN-γ and release granzymes, as well as perforin to remove infected cells and strengthen CD8^+^ T-cell response (57, 58). Accumulation of NK is found in the lung and airways dependent on the cell surface chemokine receptors, such as CC-chemokine receptor 5 (CCR5) and CXC-chemokine receptor 3 (CXCR3) (59). Activation of natural cytotoxicity receptors (NCRs) expressed on NK cells relied on the binding between NKp46 and NKp44 to viral hemagglutinin (HA) (60, 61). This kind of activation results in the lysis of infected cells mediated by NK cells through degranulation, perforin and granzyme release, and IFNγ secretion (62, 63). The function of NK cells can be influenced by ROS. It has been proved that ROS derived from monocytes alters the signaling transduction of NK by reducing CD16ζ chain to inhibit their function (64). ROS signaling is necessary for pro-inflammatory cytokine release, including type I IFN produced by NK cells. Therefore, ROS may participate in the regulation of cytokine production of NK cells responding to IAV infection, which needs to be proved by further studies.

### Adaptive immune response

The adaptive immune response is the second line of defense against pathogens. Unlike fast response by the innate immune system, the development of adaptive immune response is a highly specific and long-lasting process, which is necessary for the clearance of virus and protection against future invasion through the establishment of long-term memory (65).

#### Dendritic cells

Evidence has showed that acute infection of IAV resulted in reduced number of cDCs (CD11c^+^ conventional DCs) and pDCs (plasmacytoid DCs) in peripheral circulation, but a sustained enhancement in respiratory tract (62). The murine CD103^+^ DCs traffic viral antigen to lymph nodes and present to CD8^+^ T cells to control replication of virus (66–68); however, CD11b^+^ cDCs activated by IAV fail to prime CD8^+^ T cells (69). Massive accumulation of IFN-α produced by pDCs may contribute to uncontrolled inflammation and pathology (70). Accumulation of pDCs in lymph nodes under lethal infection upregulate the expression of Fas ligand, which recognize its receptor Fas expressed on IAV-infected CD8^+^ T cells, to promote elimination by Fas-dependent apoptosis (71, 72). Altered levels of ROS impact the antigen presenting capacity of DCs. It has reported that inhibited levels of ROS significantly decrease antigen uptake of DCs (73, 74); reduced mitochondrial ROS, to be specific, decreases antigen presentation of pDCs to CD8^+^ T cells (75); nevertheless, enhanced level of ROS suppressed antigen presentation of DCs as well by induction of mitochondrial disorder (76). DCs themselves produce ROS at a slow but prolonged manner. It was revealed that ROS could be produced by DCs within minutes and sustained for at least 10h with an average rate around 0.5mM/sec per phagosome, which was about 10-fold lower than that in neutrophils (77). ROS, on the other hand, has ability to increase the intracellular Ca^2+^ concentration by activating the transient receptor potential melastatin 2 (TRPM2) channels, which are essential for Ca^2+^-permeable and preferentially present in the lysosomal membranes in DCs (78). The release of Ca^2+^ from activated TRPM2 channel provides vital signal for the maturation and chemotaxis of DCs (78, 79). However, further investigation on the ROS-mediated function of DCs responding to IAV infection is still needed.

#### T cells

CD4 T cells are found to be correlated with lower IAV shedding and less severe outcome in infected patients (80). They can migrate to the infection sites by contacting with virus-specific antigens to activate CD8 T cells and modulate immune response mediated by virus-specific CD8 T cells (81, 82). Release of IFN-γ by CD4 Th1 cells is necessary for the development and preservation of CD8 T memory (83, 84). The function of CD4 T cells during the initial priming phase of infection limits exhaustion of CD8 T cells, which can rapidly recall the viral memory in future infection (85). CD4 CTL utilizes a perforin/granzyme-mediated mechanism to perform cytotoxicity role for elimination (86, 87). IL-2 induced Jak3 and STAT5 are required for optimal formation of CD4 CTL. A recent study revealed that STAT1 protected CD4 CTL against NK during IAV infection, and STAT4 enhanced the promotion of Th1 identity to improve their anti-viral impact (88).

CD8 T cells contribute to defense immunity against IAV infection by releasing cytotoxic granules and cytokines and inducing direct apoptosis of infected cells (89). IAV-specific CD8 T cells have been mostly enriched in the lung of patients (90) and reach the peaks of frequency at approximately day 10 after infection (91). Following infection, CD8 T cells recognize highly conserved epitopes derived from internal influenza components (92). CD8 T cells then get activated and acquire the effector ability to secret inflammatory cytokines and effector molecules. During the pandemic in 2009 caused by IAV, development of severe diseases is correlated with less frequency of virus-specific CD8 T cells (93). A subset of virus-specific CD8 T cells keep in the host to protect against further infection by forming long-lasting memory population (91). Memory subtypes can remain in human up to several months, an over 13 years presence in peripheral blood have also been found (94). The memory response to IAV in human is stable and can trigger rapid expansion in the lung towards the secondary infection (95, 96). Their expansion in the lung and airways correlates with increased CXCR3- and CCR5-binding chemokines (97). After expansion, memory subtypes produce cytokines, like IFN-γ and TNF, in a rapid manner (98, 99); meanwhile, high expression of CD11a helps them to produce cytolytic molecules to clear and protect against the virus (100). CD8 T cells express Fas ligand (CD95L) and TNF receptor apoptosis-inducing ligand (TRAIL), which can interact with their receptor Fas (CD95) and DR4 and/or DR5, respectively, to mediate apoptosis of infected cells (91).

Activation of both CD4 and CD8 T cells triggers the respiratory burst either by direct contact with phagocytes or by cytokines, meanwhile, phagocytes-generated ROS in turn impacts T cells and leads to oxidative stress. NOX-2 produced ROS have been found involved in the differentiation of T cells (101). The susceptibility to ROS strongly depends on the subtype of T cells, CD45^+^RA T naive cells are more resistant to ROS-induced apoptosis than CD45^+^RO T memory cells (102). Effector T cells are largely protected from ROS-mediated cell death (103), which may partly due to the glutathione precursor cysteine and the thiol-reducing enzyme thioredoxin released by macrophages and DCs (101). The generation of ROS plays an essential role for massive expansion of CD8 T cells responding to IAV infection (104). The metabolism of mitochondria is also critical for T-cell activation that mitochondrial ROS activated nuclear factor of activated T cells (NFAT) are necessary for IL-2 secretion (105). Meanwhile, mitochondrial ROS can be converted to hydrogen peroxide signal, which induces CD95L expression to regulate activation-induced T-cell death (AICD) (106). As modulators, ROS play an indispensable role in T-cell receptor-induced transcription. The generation of ROS constitutes an intriguing issue with multiple implications for both T-cell-activated bioenergetics and T-cell-mediated pathologies (107).

#### B cells

B cells contribute to the production of pathogen-specific antibodies to inactivate pathogens and to eliminate the infected cells (108). They also present viral antigens for downstream stimulation (109) and secrete anti- and pro-inflammatory cytokines during viral clearance (110). Following IAV exposure, naïve B cells are activated by viral antigens and differentiate either into short-lived antibody-producing plasmablasts (PBs) or germinal center (GC) B cells (111). GC is the place for affinity maturation and clonal selection of GC B cells (111, 112). Within GC, successfully mutated GC B cel clones present antigen peptides to T follicular helper (Tfh) cells *via* MHC-II (111). With the help of Tfh, the affinity of GC B cells decides their differentiate that the highest affinity is selected for long-lived plasma cells (LLPCs), while lower ones for memory B cells (MBCs) (113–115). Encountered the secondary infection, MBCs are recalled and differentiate rapidly into PBs, or re-enter into GCs for affinity mature (116–118). Simultaneously, naïve B cells are recruited again to generate a *de novo* B cell response to variate the antibody response against shared and drifted epitopes (117, 118). As a result, newly educated and generated LLPCs and MBCs fight together to protect host against new viral variant.

ROS production is involved in B cell receptor (BCR) stimulation. BCR stimulation of primary resting B cells induces a rapid generation of ROS for at least 24h in mice, in where early production of ROS (0-2h) relies on Nox2, while the later ROS (6-24h) depends on mitochondrial respiration (119). Produced ROS further stimulate BCR downstream for effective B cell response through PI3K pathway (119). A further study confirms the role of prolonged ROS in B cell proliferation (120). In mouse splenic B cells, ROS generated by BCR ligation are produced in two phases. The first one happened immediately and ceased in 1h, while the second one started at 2h and lasted for 4-6h. ROS, produced by NOX3 not NOX2 in the late phase, enhance the activation of essential pathways for B cell proliferation, including NF-κB and PI3K pathways (120).

## Discussion

Human respiratory virus infections lead to a spectrum of respiratory symptoms and disease severity, contributing to substantial morbidity, mortality and economic losses worldwide, as seen in the COVID-19 pandemic. Respiratory virus, mainly including IV, respiratory syncytial virus (RSV), MERS-related coronavirus (MERS-CoV), and severe acute respiratory syndrome coronavirus (SARS and SARS-CoV-2), show comparable symptoms in patients. In this review, we discuss the ROS involvement of immune responses to IAV infection, a representative of respiratory virus, including mitochondria function, oxidant and antioxidant enzymes, and signaling pathways in various immune cells. Extensive research has suggested that hyperinflammation induced by IAV is heavily involved with oxidative stress. Therefore, the elevated oxidative stress is commonly observed in IAV infected cases with severe disease progress (121–123). Consistently, several factors that are known to be associated with increased ROS levels and oxidative stress, such as chronic diseases, morbid obesity, smoking, and older age, are listed as risk factors for a more severe course on IAV infected patients (124–126). Severe hyperinflammation caused by high levels of ROS is spurred by an exuberant but dysregulated immune response (127). The current treatment in the clinic for hyperinflammation induced by mainly relies on immunomodulatory therapy, including broadly immunosuppressive approaches (such as glucocorticoids) (128, 129) and targeted immunomodulatory therapies (such as cytokine blockades) (130). The understanding of ROS impact on immune response will provide novel therapeutic targets to effectively treat IAV infection, and other respiratory virus infection in general.

## Author contributions

LW, ZC, ZW, JG performed the literature review. LW wrote the manuscript. JW revised the manuscript, JW and LW organized and provided the frame of this manuscript. All authors read and approved the final manuscript. All authors contributed to the article and approved the submitted version.

## Funding

This work was supported in part by R01 CA253215 (JW), the UCLA AIDS Institute, the James B. Pendleton Charitable Trust and the McCarthy Family Foundation. The funders had no role in study design, data collection and analysis, decision to publish, or preparation of the manuscript.

## Conflict of interest

JW has a financial interest in Vivibaba and The Regents have licensed intellectual property invented by JW to Vivibaba. No funding was provided by these companies to support this work.

The remaining authors declare that the research was conducted in the absence of any commercial or financial relationships that could be construed as a potential conflict of interest.

## Publisher’s note

All claims expressed in this article are solely those of the authors and do not necessarily represent those of their affiliated organizations, or those of the publisher, the editors and the reviewers. Any product that may be evaluated in this article, or claim that may be made by its manufacturer, is not guaranteed or endorsed by the publisher.
